# Co-Infection of the Epstein–Barr Virus and the Kaposi Sarcoma-Associated Herpesvirus

**DOI:** 10.3390/v14122709

**Published:** 2022-12-02

**Authors:** Michelle Böni, Lisa Rieble, Christian Münz

**Affiliations:** Viral Immunobiology, Institute of Experimental Immunology, University of Zürich, 8057 Zurich, Switzerland

**Keywords:** natural killer cells, cytotoxic lymphocytes, T cells, latent and lytic infection, B cell lymphomas, Kaposi sarcoma, humanized mice

## Abstract

The two human tumor viruses, Epstein–Barr virus (EBV) and Kaposi sarcoma-associated herpesvirus (KSHV), have been mostly studied in isolation. Recent studies suggest that co-infection with both viruses as observed in one of their associated malignancies, namely primary effusion lymphoma (PEL), might also be required for KSHV persistence. In this review, we discuss how EBV and KSHV might support each other for persistence and lymphomagenesis. Moreover, we summarize what is known about their innate and adaptive immune control which both seem to be required to ensure asymptomatic persistent co-infection with these two human tumor viruses. A better understanding of this immune control might allow us to prepare for vaccination against EBV and KSHV in the future.

## 1. Introduction to EBV and KSHV

The two human γ-herpesviruses, Epstein–Barr virus (EBV) and Kaposi sarcoma-associated herpesvirus (KSHV), are WHO class I carcinogens [1]. They are associated with lymphomas and carcinomas that fortunately only develop in a small percentage of persistently EBV and KSHV infected individuals [2,3,4,5]. EBV persists in more than 95% of the adult human population and KSHV is most frequent in Sub-Saharan Africa with a seroprevalence of more than 50% in many countries but below 10% in Northern Europe and Northern America [4,6]. Both viruses are thought to be primarily transmitted via saliva exchange, and infect B cells in submucosal secondary lymphoid tissues, such as tonsils [6,7]. EBV might cross the mucosal epithelium via transcytosis [8,9]. EBV establishes latent antigen expression after infection that drives B cells into proliferation and rescues them from cell death. This leads to B cell immortalization, as can be observed in vitro during the generation of lymphoblastoid cell lines (LCLs) by EBV infection of primary human B cells [10]. The latency III program that is found in LCLs consists of six EBV nuclear antigens (EBNA1, 2, 3A, 3B, 3C and -LP), two latent membrane proteins (LMP1 and 2), two small non-translated RNAs (EBER1 and 2) and more than 40 miRNAs. It can also be detected in naïve tonsillar B cells of healthy virus carriers [11]. In germinal center B cells, latent EBV protein expression is reduced to EBNA1, LMP1 and LMP2. This latency II program is thought to provide CD40 and B cell receptor (BCR)-like signaling to rescue infected B cells from the germinal center reaction. This differentiation allows EBV to gain access to the memory B cell pool in which all latent EBV protein expression is turned off (latency 0) or EBNA1 is transiently expressed to maintain the viral DNA in homeostatically proliferating memory B cells (latency I) [12,13]. From this reservoir of long-term persistence, EBV reactivates into lytic replication and infectious viral particle production, most likely due to BCR stimulation-induced plasma cell differentiation [14]. Accordingly, the viral transcription factor BZLF1 that initiates lytic EBV replication in B cells is induced by the plasma cell-associated transcription factors, BLIMP1 and XBP1 [15,16]. Basolateral infection of mucosal epithelial cells [8] might then allow for another round of lytic EBV replication as is pathologically observed during oral hairy leukoplakia [17] for efficient viral shedding into saliva and further transmission. Therefore, all latent EBV infection patterns that are found in B cell lymphomas, including latency I of Burkitt’s lymphoma, latency II of Hodgkin’s lymphoma and latency III that can be observed in some diffuse large B cell lymphomas (DLBCL), are already present in healthy EBV carriers. Immune suppression due to human immunodeficiency virus (HIV) co-infection or iatrogenic immune suppression after transplantation allows these premalignant states to develop into the respective lymphomas. For KSHV, the sites of latent and lytic infection are much less well defined. However, due to the emergence of Kaposi sarcoma (KS), primary effusion lymphoma (PEL) and multicentric Castleman’s disease (MCD) during immune suppression, KSHV persists and is presumably immune controlled in endothelia and B cells from which Kaposi sarcoma and the KSHV-associated lymphomas emerge [4,5]. How the three KSHV latent gene products, viral FADD-like interleukin-1-β-converting enzyme inhibitory protein (vFLIP), viral cyclin (vCyclin) and latent nuclear antigen (LANA), and its lytic gene products contribute to the non-pathogenic cellular reservoirs of persistent KSHV infection remains to be defined. However, recent studies suggest that at least some of these benefit from co-infection by EBV for KSHV persistence.

## 2. Persistence of KSHV in EBV Infected B Cells

KSHV infection has been associated with primary effusion lymphomas (PELs) since 1995, and KSHV detection has been an important part of the PEL diagnosis ever since [18,19]. Knockdown of LANA as well as vCyclin and vFLIP has led to growth inhibition and apoptosis in PEL cell lines, and leads to a reduction in KSHV genome levels [20]. Further, knockdown of the viral interferon regulatory factor 3 (vIRF3) has also been shown to reduce proliferation of PEL cells and increase apoptosis levels [21]. All this supports the association of PEL with KSHV infection.

In addition to KSHV, about 90% of PELs show persistence of EBV [22,23,24]. Co-infection is frequently detected in established PEL cell lines, with both viral genomes maintained and independently replicated and partitioned to the daughter cells [22,25,26]. In vitro studies showed that KSHV alone can infect but not transform peripheral B cells and therefore cannot persist long term [27,28,29]. In vivo dual-infection studies in mice with reconstituted human immune system components (humanized mice) have added evidence that co-infection with EBV increases the probability of KSHV persistence [30,31]. Co-infection with EBV activates B cells and supports long-term KSHV infection and cell proliferation through transformation depending on expression of at least one transforming EBV gene [25,27]. Persistence of KSHV is not dependent on EBV lytic gene expression, as KSHV can also persist in cells infected with an EBV BZLF1 knockout virus that lacks lytic gene expression in vitro and in vivo [30].

B cell transformation has been shown to be dependent on five viral latent antigens, namely EBNA2, EBNA-LP, EBNA3A, EBNA3C and LMP1. Proliferation of infected cells is initiated by EBNA2 through expression of cell cycle genes such as c-myc and cyclins D2 and E [32,33]. EBNA-LP is reported to enhance this EBNA2-induced gene activation [34,35]. EBNA3A and EBNA3C block the DNA damage response; however, animal experiments have shown that they are not necessary for EBV persistence [32,33,36]. LMP1 expression contributes to transformation and proliferation as well as cell survival by engaging NF-κB signaling pathways and mimicking CD40 signaling [37,38,39]. EBV LMP1 supports latency establishment through inhibition of lytic replication, and transcriptional control in PEL allows for sporadic expression of LMP1 and non-coding RNAs [40,41,42,43,44,45,46]. Expression of these gene products creates conditions permitting KSHV persistent infection and PEL emergence [27].

The link of EBV and PEL proliferation is further supported by the fact that EBV genome loss reduces both KSHV genome maintenance and proliferation [25,27,47]. The exact mechanism is unknown, but EBV co-infection seems to maintain KSHV genomes, as evident by the increased amount of KSHV genomes per cell observed in co-infected cells [25,27,30,48]. Later during co-infection, expression of both EBV and KSHV is restricted to a reduced latent EBV gene expression. It is mostly restricted to EBNA1 and non-translated RNAs (latency I), as KSHV LANA induces methylation and silencing of the major latent promoters Qp and Cp that regulates expression of EBV latency III genes [49].

While EBNA1 might only contribute little to B cell transformation, loss of its expression in EBV^+^ KSHV^+^ PEL cell lines reduced proliferation, indicating a role of EBNA1 in the promotion of KSHV persistence and B cell growth [40,47]. 

Aside from EBV genes, PEL cells depend on latent KSHV gene expression, mainly LANA, vFLIP and vIRF3 for survival, as they interact with tumor suppressors and inhibit apoptotic processes [20,21,50,51,52]. LANA mediates the persistence of the KSHV episome by interaction with KSHV terminal repeat sequences [53,54]. This persistence does not depend on further viral genes, and episomes are lost upon LANA knockdown [55]. LANA also mediates replication of the episomal DNA and tethers the virus DNA to host mitotic chromosomes, facilitating division of the KSHV genome to the daughter cells [56]. vFLIP can activate NF-κB, which is constitutively active in PEL [57,58,59]. It averts FAS-induced apoptosis through interaction with the death-inducing signaling complex (DISC) that prevents processing of procaspase 8 [60]. vIRF3 is required for survival of both EBV^+^ and EBV^−^ PEL as knockdown lead to an increase in apoptosis and reduced proliferation [21]. 

Apart from these molecular interactions of EBV and KSHV gene products for persistence of both viruses in B cells in vitro and in mouse models, epidemiological evidence in Sub-Saharan Africa has suggested that KSHV infection is nearly uniformly associated with EBV co-infection and that EBV seropositivity is among the strongest environmental risk factors for KSHV seropositivity [61,62]. Therefore, EBV gene expression contributes to the persistence of KSHV in B cells, promoting B cell transformation, proliferation and survival. This allows for KSHV persistence due to EBV co-infection in vitro, in mouse models and in a human African patient cohort.

## 3. Primary Effusion Lymphomagenesis Due to EBV and KSHV Co-Infection

As EBV increases KSHV persistence, KSHV genome copy numbers per cell and cell proliferation, it is highly likely that it also impacts primary effusion lymphomagenesis. Development of primary effusion lymphoma is still not completely understood, but in vitro and recent in vivo studies suggest a role of viral lytic gene expression in driving tumorigenesis [30,63].

**Figure 1:**
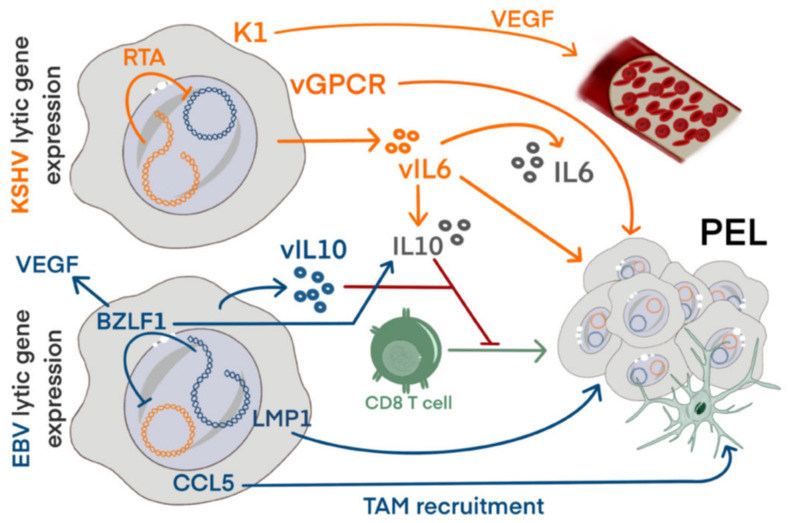
Expression of lytic EBV and KSHV genes can condition the tumor microenvironment. Primary effusion lymphoma (PEL) is associated with KSHV, however 90% of tumors also carry EBV. EBV and KSHV most likely contribute to the tumor environment simultaneously through their lytic gene expression. Lytic KSHV expression contributes through expression of K1, which promotes expression of VEGF and angiogenesis. viral G-protein coupled receptor expression promotes proliferation. Expression of the viral cytokine vIL6 promotes production of IL6 and IL10 and increases PEL proliferation. EBV lytic gene expression contributes through CCL5 production that attracts monocytes, which as tumor associated macrophages (TAM) have immune suppressive functions. Expression of viral IL10 can suppress CD8^+^ T cell responses.

EBV and KSHV dual-infected humanized mice present with increased lymphomagenesis and enhanced levels of early EBV lytic gene expression [30,64,65]. These enhanced levels of EBV lytic gene expression are also detected in co-infected PELs, supporting the role of lytic genes in tumorigenesis [16,30,66,67]. Infection with BZLF1-deficient EBV demonstrated a reduction in lymphoma formation, whereas infection with an EBV variant that increases lytic replication demonstrated increased lymphomagenesis compared to EBV wildtype infection in humanized mice [64,68,69]. It is likely that the increase in tumor formation is promoted by abortive lytic EBV expression, as full lytic EBV reactivation would rather decrease tumor formation by the destruction of infected cells during the production of new viral particles [6,70]. Expression of BZLF1 induces lytic gene expression as well as the expression of immune evasins and proteins protecting the cells from apoptosis [71]. BZLF1 itself has been shown to play a prominent role in tumor progression through its capability to induce VEGF and IL10 secretion (Figure 1), supporting vascularization and suppressing T cell responses [72,73,74,75,76]. Lack of the late lytic gene BALF5 increases establishment of lymphomas from transformed B cells in immunocompromised mice, confirming a role of early lytic genes [77]. In EBV^+^ B cells, tumor necrosis factor (TNF), CCL5 and IL10 expression is increased upon spontaneous lytic reactivation [78,79,80]. This links lytic EBV expression to conditioning of the tumor microenvironment, as TNF is involved in inflammation and immune regulation, CCL5 is important in the recruitment of myeloid suppressor cells and IL10 suppresses T cell responses [78,79,80]. Adding to this, EBV itself encodes for a viral homologue of IL10 (vIL10) [81]. KSHV encodes for a viral homologue of IL6 (vIL6) that in turn can upregulate production of human IL6 and IL10 [82]. vIL6 cooperates with c-myc and drives formation of plasmablastic neoplasms in immunocompromised mice, as well as it increased the number of tumors in a murine xenograft model and supported metastasis [83,84,85,86]. These cytokines increase the production of Vascular Endothelial Growth Factor (VEGF) and together, this promotes proliferation, cell survival, immunosuppression, neoangiogenesis and activation of oncogenic signaling pathways such as the NF-κB pathway [82,87,88,89,90]. 

Many studies demonstrate an important role for a multiplicity of KSHV genes in lymphomagenesis. ORF36, a viral protein kinase, leads to increased hyperproliferation of B cells as well as lymphoma development [91]. Transgenic expression of the transmembrane glycoprotein K1 promotes lymphoproliferations that show NF-κB activation [92,93]. K1 can also induce expression of VEGF and pro-inflammatory cytokines like IL6, IL8 and IL10 [93,94,95]. Viral G-protein coupled receptor (vGPCR) increases expression of pro-inflammatory cytokines and contributes to tumor formation that resembles Kaposi sarcoma when expression is induced in mice [96,97,98,99,100]. 

vIRF3 drives an oncogenic transcriptional program mediated by super-enhancers through cooperation with cellular IRF4 and BATF [21,43]. RTA, the replication and transcription activator of KSHV, can transactivate EBV latency promoters by complexing with RBP-Jκ [44]. This cooperation induces LMP1 expression in an EBV latency I background, contributing to cell growth that is EBV-driven [44]. It further interacts with the EBV lytic inducer BZLF1, inhibiting EBV lytic gene expression [44,45,101]. LMP1, in turn, contributes to tumor formation through inducing expression of the oncogenic protein UCH-L1 [102]. The latent KSHV gene LANA has also been shown to induce UCH-L1, and co-infection has shown that LANA and LMP1 synergize to activate UCH-L1, promoting a tumorigenic phenotype with an increase in proliferation, adhesion, cell migration and apoptosis inhibition [102]. 

This evidence shows that both EBV and KSHV contribute to the primary effusion lymphomagenesis and co-infection can increase the likelihood of tumor formation by shaping the tumor microenvironment and providing proliferation and survival advantages.

## 4. Modulation of Innate Immune Responses by EBV and KSHV

**Figure 2:**
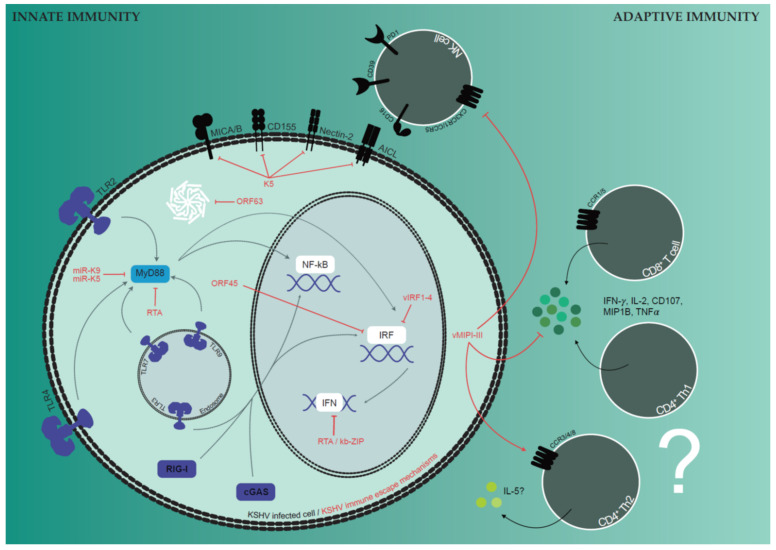
Balance between host immune responses and viral immune modulation mechanisms allow persistence of KSHV. TLR, RLR, NLR and intracellular DNA-sensor cGAS are the four PRRs reported to sense KSHV infection (blue) and to induce NF-κB-mediated inflammatory cytokine production, type I IFN response and inflammasome activation (white). KSHV immune evasions (red) counteract PRR-induced signaling pathways via different means, e.g., via reducing the expression of signaling proteins (miR-K9/K5, RTA, kb-ZIP), via suppression of cellular proteins by viral homologues (vIRF1-4, ORF63), via targeting signaling proteins for proteasomal degradation (RTA) or via inhibition of nuclear translocation of signaling proteins (ORF45). Cellular innate immune response is modulated by reducing cytotoxicity of NK cells via driving differentiation into a late phenotype characterized by CD39 expression and loss of NKG2D, via downregulation of activating NK cell receptor ligands and via inhibiting NK cell migration by viral chemokine secretion. IFN-γ derived from NK cells, CD8^+^ or Th1 CD4^+^ T cells might protect from KSHV-associated malignancies, although T cell correlates conferring protection from KSHV-associated malignancies are not fully understood.

Human γ-herpesviruses, unlike viruses that only achieve acute infections, are not cleared by human immune responses, and establish latent infections [6,103]. It is a fine-tuned balance between the host immune responses and the pathogen immune evasion mechanisms that allows this persistence of EBV and KSHV without causing disease. This equilibrium, in which KSHV and EBV modulate the observed immune responses, was established during co-evolution over time and can be recognized in both innate and adaptive immunity to these viruses. Focusing on innate immunity, four classes of pathogen recognition receptors (PRR) are reported to be implicated in the recognition of EBV and KSHV: Toll-like receptors (TLR), RIG-I-like receptors (RLR), NOD-like receptors (NLR) and intracellular DNA-sensors like cGAS [104,105,106]. Activation of these pathways primarily leads to NF-κB-mediated production of inflammatory cytokines, induction of type I interferons (IFNs) or inflammasome activation and can be mediated by infected cells such as B cells, plasmacytoid dendritic cells (pDCs) and endo- and epithelial cells themselves. Apart from infected cells, activated monocytes, macrophages and classical dendritic cells (cDCs) harboring those PRR can also induce such responses. Despite knowledge of the involved pathways, there is no primary immunodeficiency (PID) affecting type I IFN responses described to predispose for γ-herpesviruses, and there remains a lot of open questions on how the innate immune sensing of both viruses influences the course of infection [107,108,109,110,111]. Along this line, it was shown that in vivo depletion of pDCs, even though being the main source of IFN after EBV infection, had only transient effects on EBV infection or on CD8^+^ T cell responses, which were thought to be primed by DCs [107]. Furthermore, pDCs are transiently depleted during symptomatic primary EBV infection in humans [112,113]. This insensitivity to type I IFN responses might be caused by the plethora of gene products of all γ-herpesviruses counteracting the above-mentioned immune responses reviewed in detail by Lange et al., stressing the importance to overcome early defense mechanisms for persistent infections [105]. In general, similar strategies are applied by both EBV and KSHV (Figure 2), all leading to the inhibition of PRR-mediated responses. In the first place, viral gene products may interfere with the expression of host proteins involved in PRR signaling cascades, either directly via viral miRNAs, by possessing exonuclease activity or by interacting with promoter sites to inhibit anti-viral gene expression [114,115,116,117,118,119]. So, for example, KSHV miRNA miR-K9 and miR-K5 can directly target MyD88, leading to reduced pro-inflammatory cytokine production and both KSHV LANA and kb-ZIP can abrogate IFN-*β* promoter activity [115,116,120]. Next, cellular proteins can be suppressed by expression of viral homologues, such as KSHV vIRF1-4 inhibiting the cellular interferon regulatory factors or KSHV ORF63 inhibiting inflammasome activation by NLRP1 mimicry [89,121,122,123]. In addition, viral phosphatases and kinases such as EBV BGLF4 can directly modulate enzyme activities thereby decreasing PRR downstream signaling [124,125,126]. In addition, viral proteins can modulate ubiquitylation and proteasomal degradation, exemplified by KSHV RTA, which possesses E3-ubiquitin ligase activity and targets, for example, MyD88 [127,128]. Finally, the direct interaction of gene products for both virus and host can prevent conformational changes or nuclear translocation, as it is the case for KSHV ORF45, which blocks the nuclear translocation of IRF7 [129,130]. Besides expressing viral immune evasions, the inexistence of protein expression of the EBV latency program 0 and the low expression level of all latent EBV proteins can be regarded as a hiding mechanism from human immune responses [104]. Overall, it still remains unclear if the viral pattern recognition in infected cells or bystander antigens present or viral sensing dendritic cells restrict EBV and KSHV infection. 

An additional line of early defense is mediated by innate immune cells such as NK, NKT and γδ T cells, whose phenotype might be directly shaped by the viral infection. Underlining the importance of NK cell responses in EBV infection are PIDs affecting NK cell differentiation, activating NK cell receptors or NK cell effector functions, but also the observed expansion of NK cells during infectious mononucleosis (IM) with regards to numbers and frequency [131,132,133,134]. Expanding NK cells are in an early differentiation state; CD56^dim^CD16^+/−^NKG2A^+^NKG2C^−^ and their protective function might be mediated either via activating NK cell receptors NKG2D and DNAM-1, via CD16-mediated antibody-dependent cellular cytotoxicity targeting lytically-replicating EBV, or via preventing B cell infection by direct removal of viral particles bound to the B cell surface [132,135,136,137,138]. Further differentiation driven by co-infection, in case of CMV into NKG2C^+^KIR^+^ adaptive NK cells, was shown to go along with impaired EBV-specific immune control [139]. Similarly, co-infection with KSHV is associated with further NK cell differentiation into CD56^−^CD16^+^CD39^+^ NK cells in humanized mice, an even less cytotoxic phenotype that might suppress immune responses via CD39 [31]. This reduced NK cell cytotoxicity is also observed in KS patients, which correlates with downregulated-activating NK cell receptors such as NKG2D, NKp30 or CD161 and with upregulation of the inhibitory receptor PD-1 [140,141,142]. Furthermore, KSHV gene products directly protect the infected cells by downregulating activating NK cell receptor ligands on their surface such as NKG2D ligands MICA/B, AICL, CD155 or Nectin-2 but also via secreting the viral chemokine vMIP-II blocking NK cell receptors involved in NK cell migration, such as CX3CR1 and CCR5 [143,144,145,146,147]. Therefore, early differentiated NK cells restrict lytic EBV infection, but KSHV co-infection compromises the cytotoxic function of these innate lymphocytes.

## 5. Adaptive Immune Responses to EBV and KSHV

Imbalance between host and pathogen can also be caused by deviations in the adaptive immune response and may lead to diseases such as IM in cases of overactive immune responses or to the development of malignancies or chronic active EBV in cases of lacking immune responses. Characteristics of patients affected by γ-herpesvirus associated malignancies include impaired cytotoxic responses, especially T cell responses [148]. Reasons may be primary immunodeficiencies (PID) affecting TCR signaling, costimulatory molecules and IFNγ signaling, but also co-infection with HIV, iatrogenic immunosuppression or advanced age [22,131].

The main cytotoxic effectors, the CD8^+^ T cells, highly expand in numbers during IM, the acute symptomatic primary EBV infection [133,149]. In IM, single EBV specificities can make up to 50% of the total CD8^+^ T cells during IM [150,151,152]. EBV specific T cells are primarily directed against immediate early (IE) gene products, to a lesser degree against early (E) gene products and even fewer against late (L) gene products, while latent antigen-specific T cells only make up around 0.1–0.5% and are mainly directed against the EBNA3 family of proteins [152,153,154,155]. The hierarchy of recognized antigens also remains during latent infection, although upon contraction of T cell numbers, the frequencies of EBV-specific CD8^+^ T cells decrease to 2% recognizing lytic and to 1% recognizing latent gene products, respectively [155,156,157,158]. The expanded CD8^+^ T cells during IM are of an activated phenotype being HLA-DR^+^CD38^+^CD69^+^Ki-67^+^ but lacking lymphoid homing markers such as CCR7 or CD62L, thus potentially explaining the low recruitment into tonsils resulting in lower EBV-specific T cell responses at the site of infection [150,154,159]. CD4^+^ T cells do not expand in numbers, yet EBV-specific responses increase to up to 1% of total CD4^+^ T cells and thereby contribute to increased overall activation of CD4^+^ T cells [133,160]. Contrary to CD8^+^ T cells, they are more often directed against latent antigens and may emerge delayed with EBNA1-directed responses arising only several months after primary infection [160]. EBV-directed CD4^+^ T cells can be cytotoxic and are mostly of a Th1-like phenotype expressing T-bet, IFN-γ, TNFα, Perforin and Granzyme B [161,162,163,164]. During asymptomatic primary infection, similarly high viral load levels as in IM patients were detected in a cohort of Gambian children, though without the accompanying CD8^+^ T cell expansion, questioning the protective effect of these cells during early years of life when seroconversion often occurs [165]. Nevertheless, successful adoptive transfer experiments of EBV-specific CD8^+^ T cells in lymphoma and PTLD patients, and depletion experiments in humanized mice leading to increased lymphomagenesis underline the protective value of EBV-specific CD8^+^ T cells [166,167]. Those lines of evidence are absent for KSHV-specific immunity. Epidemiology and PID predisposing for KSHV-associated diseases speak strikingly for an involvement of T cells, but experimental data are scarce [22,131]. In the early 21st century, substantial effort was put into identifying targeted epitopes, but only recent studies by Roshan and Nalwoga systematically investigated KSHV-directed IFN-γ responses on a proteome-wide scale [168,169,170]. Both studies showed very weak KSHV-directed T cell responses around 1 log lower in magnitude compared to EBV and CMV controls, and high heterogeneity between patients with no immunodominant antigen being recognized by most individuals. In addition, the amount of recognized KSHV antigen derived peptide pools of 1–5 per individuum differs greatly from EBV infection with a mean of 21 different proteins recognized per patient [155]. Earlier reported work on the predominant recognition of early and late lytic KSHV-antigens was based on trends seen in seven individuals only and does not seem to be confirmed in the study by Nalwoga et al. [168,171]. The hierarchy observed in responses towards EBV antigens might have to do with the direct priming of T cells by infected B cells and with evasions expressed in late lytic stages which simultaneously reduces the presentation of those genes, making it more unlikely to be recognized by T cells [155,172]. This leaves room for speculations that the lack of hierarchy in KSHV-directed responses might be a hint towards cross-primed responses that could be initiated by dendritic cells. 

In contrast to EBV, there is no severe or prototypic illness associated with primary KSHV infection allowing for the characterization of protective immune correlates [170,173,174]. Even though there are cases described in which mononucleosis or lymphadenopathy were associated with acute or reactivated KSHV infection, most reported patients suffer only from mild symptoms such as rashes or fever which are, contrary to EBV-related IM, not accompanied by a massive cytotoxic T cell expansion [173,174,175,176]. One of the first prospective studies characterizing the immune composition upon KSHV seroconversion showed no changes in T cell numbers or in phenotype, but occurrence of KSHV-directed IFN-γ responses along with KSHV viremia [173]. T cell responses seemed to peak only 1–2 years after seroconversion. Focusing more on chronic KSHV infection, another study observed no changes in *αβ* T cell subset frequencies, but a higher frequency of *γδ* V*δ*1 T cells in KSHV^+^HIV^−^ individuals compared to age-matched KSHV^−^ controls [177]. These *γδ* V*δ* T cells were strongly reactive against KSHV-infected PEL cell lines, which contrasts to what was observed for *αβ* T cells: in vitro experiments using CTL clones or Jurkat cells showed that PEL cell lines elicit only weak T cell responses, probably due to the interference of KSHV with MHC class I and II restricted antigen presentation [178,179,180]. However, implications of the impaired immunogenicity of PEL cell lines in a clinical setting remain unclear since most studies focusing on KSHV-directed T cell responses do not specifically focus on PEL patients but only differentiate between healthy and diseased virus carriers, including MCD and KS patients. Even there, data are somewhat contradictory with Roshan et al. reporting greater diversity in recognized antigens in diseased patients and earlier studies from Guihot and Lambert reporting the opposite with a greater diversity in healthy patients [169,181,182]. Nevertheless, all three studies demonstrated that in vitro, KSHV-restricted CD4^+^ and CD8^+^ T cells derived from healthy volunteers and diseased patients can be both mono- or polyfunctional, expressing IFN-γ, IL-2, CD107, MIP-1B and TNFα [169,183,184,185]. This cytokine profile is in agreement with PIDs affecting IFN-γ receptor or STAT4 that predispose for KS, with a KS tumor microenvironment in which PBMCs secrete high levels of Th1 cytokines, and also with KSHV encoded viral homologues of cellular chemokines, such as vMIPI-III, which counteract Th1 responses by rather skewing into a more Th2-like microenvironment for immune evasion [146,186,187,188,189,190]. On the other hand, the Th2-cytokine IL-5 was reported to be associated with better outcomes in KS, and pulmonary KS was shown to be associated with reduced expression of IFN-γ and other polyfunctional effectors mentioned before, therefore resulting in a reduced proinflammatory environment [191,192]. These discrepancies illustrate that the actual immune correlates conferring protection from KSHV-associated malignancies are still not identified, and while a number of studies have focused on CD8^+^ T cell-mediated IFN-γ responses, there are only a few studies investigating the importance of CD4^+^ or γδ T cell responses in KSHV infection.

## 6. Conclusions and Outlook

These immune responses against EBV and KSHV ensure co-existence without pathology in most persistently infected individuals. Therefore, it should be possible to re-establish immune control by vaccination in patients who suffer from EBV- and KSHV-associated pathologies or are at risk for these. The global disease burden of EBV- and KSHV-associated diseases, with yearly tumor incidences of 300,000 and 100,000, respectively, indeed argues for the development of EBV- and KSHV-specific vaccines [7,193,194]. Many of the respective vaccine efforts focus on the induction of neutralizing antibodies against EBV and KSHV [195,196,197,198,199,200]; even so, natural immunity is thought to be primarily mediated by cytotoxic lymphocytes [131,201]. Unfortunately, the recombinant viral vector vaccines to induce cytotoxic CD8^+^ T cell responses against EBV seem to be falling behind the neutralizing antibody-inducing vaccine efforts [202,203,204,205]. Nevertheless, an EBV-targeting vaccine will probably come into existence in the next few years and we will see how this can influence global disease burden by this human tumor virus.

Previously, it was shown that induction of neutralizing antibodies against EBV gp350, the vial envelope protein that mediates attachment via complement receptors (e.g., CD21) to human B cells, reduced the incidence of symptomatic primary EBV infection (infectious mononucleosis) by 78% [198,200]. Therefore, adolescents still seronegative for EBV and with a high risk to develop IM upon EBV infection [206,207] could benefit from a neutralizing antibody-inducing vaccine against EBV, if primary infection is thereby rendered asymptomatic and not only delayed. An increased risk for EBV-associated Hodgkin’s lymphoma and the autoimmune disease, multiple sclerosis (MS), has been observed after IM [208,209,210]. Multiple sclerosis affects more than 2 million individuals worldwide [211]. Therefore, vaccine-induced EBV neutralizing antibodies could reduce these risks for EBV-associated diseases at the same time as IM. However, in comparison to the 32-fold increased risk for multiple sclerosis by EBV infection in general [212], the 2-fold increased risk after IM compared to asymptomatic primary infection is rather modest. Nevertheless, a better understanding of the mechanistic contribution of EBV infection to MS development would enable us to assess if EBV-specific vaccination could influence this autoimmune disease. As EBV also seems to contribute to KSHV persistence and KSHV-associated tumor burden in the case of PEL, vaccination against EBV might also prove beneficial with regards to KSHV infection. KSHV-specific vaccination efforts might also significantly reduce KSHV-associated disease burden [7]. Low prevalence of this tumor virus in Middle and Northern Europe as well as North America might suggest that establishing robust immunity against KSHV by vaccination could achieve low prevalence of KSHV in Sub-Saharan Africa and Southern Europe. One would predict that this would significantly reduce the disease burden by KSHV. Therefore, robust immune control in most EBV and KSHV carriers suggests that vaccines should be developed that reinstate this immune control in patients who suffer from diseases that are associated by these two oncogenic human γ-herpesviruses.

## Data Availability

Not applicable.

## References

[1] De Martel C., Georges D., Bray F., Ferlay J., Clifford G.M. (2020). Global burden of cancer attributable to infections in 2018: A worldwide incidence analysis. Lancet Glob. Health.

[2] Shannon-Lowe C., Rickinson A. (2019). The global landscape of EBV-associated tumors. Front. Oncol..

[3] Oksenhendler E., Meignin V. (2022). HHV-8 associated lymphoma. Curr. Opin. Oncol..

[4] Cesarman E., Damania B., Krown S.E., Martin J., Bower M., Whitby D. (2019). Kaposi sarcoma. Nat. Rev. Dis. Primers.

[5] Cesarman E., Chadburn A., Rubinstein P.G. (2022). KSHV/HHV8-mediated hematologic diseases. Blood.

[6] Münz C. (2019). Latency and lytic replication in the oncogenesis of the Epstein Barr virus. Nat. Rev. Micobiol..

[7] Casper C., Corey L., Cohen J.I., Damania B., Gershon A.A., Kaslow D.C., Krug L.T., Martin J., Mbulaiteye S.M., Mocarski E.S. (2022). KSHV (HHV8) vaccine: Promises and potential pitfalls for a new anti-cancer vaccine. NPJ Vaccines.

[8] Tugizov S.M., Berline J.W., Palefsky J.M. (2003). Epstein-Barr virus infection of polarized tongue and nasopharyngeal epithelial cells. Nat. Med..

[9] Tugizov S.M., Herrera R., Palefsky J.M. (2013). Epstein-Barr virus transcytosis through polarized oral epithelial cells. J. Virol..

[10] Farrell P.J. (2019). Epstein-Barr virus and cancer. Annu. Rev. Pathol..

[11] Babcock J.G., Hochberg D., Thorley-Lawson A.D. (2000). The expression pattern of Epstein-Barr virus latent genes in vivo is dependent upon the differentiation stage of the infected B cell. Immunity.

[12] Babcock G.J., Decker L.L., Volk M., Thorley-Lawson D.A. (1998). EBV persistence in memory B cells in vivo. Immunity.

[13] Hochberg D., Middeldorp J.M., Catalina M., Sullivan J.L., Luzuriaga K., Thorley-Lawson D.A. (2004). Demonstration of the Burkitt’s lymphoma Epstein-Barr virus phenotype in dividing latently infected memory cells in vivo. Proc. Natl. Acad. Sci. USA.

[14] Laichalk L.L., Thorley-Lawson D.A. (2005). Terminal differentiation into plasma cells initiates the replicative cycle of Epstein-Barr virus in vivo. J. Virol..

[15] McDonald C., Karstegl C.E., Kellam P., Farrell P.J. (2010). Regulation of the Epstein-Barr virus Zp promoter in B lymphocytes during reactivation from latency. J. Gen. Virol..

[16] Reusch J.A., Nawandar D.M., Wright K.L., Kenney S.C., Mertz J.E. (2015). Cellular differentiation regulator BLIMP1 induces Epstein-Barr virus lytic reactivation in epithelial and B cells by activating transcription from both the R and Z promoters. J. Virol..

[17] Greenspan J.S., Greenspan D., Webster-Cyriaque J. (2016). Hairy leukoplakia; lessons learned: 30-plus years. Oral. Dis..

[18] Cesarman E., Chang Y., Moore P.S., Said J.W., Knowles D.M. (1995). Kaposi’s sarcoma-associated herpesvirus-like DNA sequences in AIDS-related body-cavity-based lymphomas. N. Engl. J. Med..

[19] Baidoun F., Moustafa M.A., Tun H.W., Hill B.T. (2022). Clinical characteristics and survival outcomes of primary effusion lymphoma: A national cancer database study. Clin. Lymphoma Myeloma Leuk..

[20] Godfrey A., Anderson J., Papanastasiou A., Takeuchi Y., Boshoff C. (2005). Inhibiting primary effusion lymphoma by lentiviral vectors encoding short hairpin RNA. Blood.

[21] Wies E., Mori Y., Hahn A., Kremmer E., Sturzl M., Fleckenstein B., Neipel F. (2008). The viral interferon-regulatory factor-3 is required for the survival of KSHV-infected primary effusion lymphoma cells. Blood.

[22] Cesarman E. (2014). Gammaherpesviruses and lymphoproliferative disorders. Annu. Rev. Pathol..

[23] Narkhede M., Arora S., Ujjani C. (2018). Primary effusion lymphoma: Current perspectives. OncoTargets Ther..

[24] Nador R.G., Cesarman E., Chadburn A., Dawson D.B., Ansari M.Q., Sald J., Knowles D.M. (1996). Primary effusion lymphoma: A distinct clinicopathologic entity associated with the Kaposi’s sarcoma-associated herpes virus. Blood.

[25] Bigi R., Landis J.T., An H., Caro-Vegas C., Raab-Traub N., Dittmer D.P. (2018). Epstein-Barr virus enhances genome maintenance of Kaposi sarcoma-associated herpesvirus. Proc. Natl. Acad. Sci. USA.

[26] Sugden A.U., Hayes M., Sugden B. (2021). How Epstein-Barr virus and Kaposi’s sarcoma-associated herpesvirus are maintained together to transform the same B-cell. Viruses.

[27] Faure A., Hayes M., Sugden B. (2019). How Kaposi’s sarcoma-associated herpesvirus stably transforms peripheral B cells towards lymphomagenesis. Proc. Natl. Acad. Sci. USA.

[28] Renne R., Blackbourn D., Whitby D., Levy J., Ganem D. (1998). Limited transmission of Kaposi’s sarcoma-associated herpesvirus in cultured cells. J. Virol..

[29] Mesri E.A., Cesarman E., Arvanitakis L., Rafii S., Moore M.A., Posnett D.N., Knowles D.M., Asch A.S. (1996). Human herpesvirus-8/Kaposi’s sarcoma-associated herpesvirus is a new transmissible virus that infects B cells. J. Exp. Med..

[30] McHugh D., Caduff N., Barros M.H.M., Rämer P., Raykova A., Murer A., Landtwing V., Quast I., Styles C.T., Spohn M. (2017). Persistent KSHV infection increases EBV-associated tumor formation in vivo via enhanced EBV lytic gene expression. Cell Host Microbe.

[31] Caduff N., McHugh D., Rieble L., Forconi C.S., Ong’echa J.M., Oluoch P.O., Raykova A., Murer A., Böni M., Zuppiger L. (2021). KSHV infection drives poorly cytotoxic CD56 negative natural killer cell differentiation in vivo upon KSHV/EBV dual infection. Cell Rep..

[32] Murer A., McHugh D., Caduff N., Kalchschmidt J.S., Barros M.H., Zbinden A., Capaul R., Niedobitek G., Allday M.J., Chijioke O. (2018). EBV persistence without its EBNA3A and 3C oncogenes in vivo. PLoS Pathog..

[33] Nikitin P.A., Yan C.M., Forte E., Bocedi A., Tourigny J.P., White R.E., Allday M.J., Patel A., Dave S.S., Kim W. (2010). An ATM/CHK2-mediated DNA damage-responsive signaling pathway suppresses Epstein-Barr virus transformation of primary human B cells. Cell Host Microbe.

[34] Saha A., Robertson E.S. (2019). Mechanisms of B-cell oncogenesis induced by Epstein-Barr virus. J. Virol..

[35] Szymula A., Palermo R.D., Bayoumy A., Groves I.J., Ba Abdullah M., Holder B., White R.E. (2018). Epstein-Barr virus nuclear antigen EBNA-LP is essential for transforming naive B cells, and facilitates recruitment of transcription factors to the viral genome. PLoS Pathog..

[36] Allday M.J., Bazot Q., White R.E. (2015). The EBNA3 family: Two oncoproteins and a tumour suppressor that are central to the biology of EBV in B cells. Curr. Top. Microbiol. Immunol..

[37] Dawson C.W., Tramountanis G., Eliopoulos A.G., Young L.S. (2003). Epstein-Barr virus latent membrane protein 1 (LMP1) activates the phosphatidylinositol 3-kinase/AKT pathway to promote cell survival and induce actin filament remodeling. J. Biol. Chem..

[38] Zimber-Strobl U., Kempkes B., Marschall G., Zeidler R., van Kooten C., Banchereau J., Bornkamm G.W., Hammerschmidt W. (1996). Epstein-Barr virus latent membrane protein (LMP1) is not sufficient to maintain proliferation of B cells but both it and activated CD40 can prolong their survival. EMBO J..

[39] Luftig M., Yasui T., Soni V., Kang M.S., Jacobson N., Cahir-McFarland E., Seed B., Kieff E. (2004). Epstein-Barr virus latent infection membrane protein 1 TRAF-binding site induces NIK/IKK alpha-dependent noncanonical NF-kappaB activation. Proc. Natl. Acad. Sci. USA.

[40] Horenstein M.G., Nador R.G., Chadburn A., Hyjek E.M., Inghirami G., Knowles D.M., Cesarman E. (1997). Epstein-Barr virus latent gene expression in primary effusion lymphomas containing Kaposi’s sarcoma-associated herpesvirus/human herpesvirus-8. Blood.

[41] Callahan J., Pai S., Cotter M., Robertson E.S. (1999). Distinct patterns of viral antigen expression in Epstein-Barr virus and Kaposi’s sarcoma-associated herpesvirus coinfected body-cavity-based lymphoma cell lines: Potential switches in latent gene expression due to coinfection. Virology.

[42] Szekely L., Chen F., Teramoto N., Ehlin-Henriksson B., Pokrovskaja K., Szeles A., Manneborg-Sandlund A., Lowbeer M., Lennette E.T., Klein G. (1998). Restricted expression of Epstein-Barr virus (EBV)-encoded, growth transformation-associated antigens in an EBV- and human herpesvirus type 8-carrying body cavity lymphoma line. J. Gen. Virol..

[43] Manzano M., Gunther T., Ju H., Nicholas J., Bartom E.T., Grundhoff A., Gottwein E. (2020). Kaposi’s sarcoma-associated herpesvirus drives a super-enhancer-mediated survival gene expression program in primary effusion lymphoma. mBio.

[44] Spadavecchia S., Gonzalez-Lopez O., Carroll K.D., Palmeri D., Lukac D.M. (2010). Convergence of Kaposi’s sarcoma-associated herpesvirus reactivation with Epstein-Barr virus latency and cellular growth mediated by the NOTCH signaling pathway in coinfected cells. J. Virol..

[45] Xu D., Coleman T., Zhang J., Fagot A., Kotalik C., Zhao L., Trivedi P., Jones C., Zhang L. (2007). Epstein-Barr virus inhibits Kaposi’s sarcoma-associated herpesvirus lytic replication in primary effusion lymphomas. J. Virol..

[46] Groves A.K., Cotter M.A., Subramanian C., Robertson E.S. (2001). The latency-associated nuclear antigen encoded by Kaposi’s sarcoma-associated herpesvirus activates two major essential Epstein-Barr virus latent promoters. J. Virol..

[47] Mack A.A., Sugden B. (2008). EBV is necessary for proliferation of dually infected primary effusion lymphoma cells. Cancer Res..

[48] Hamoudi R., Diss T.C., Oksenhendler E., Pan L., Carbone A., Ascoli V., Boshoff C., Isaacson P., Du M.Q. (2004). Distinct cellular origins of primary effusion lymphoma with and without EBV infection. Leuk. Res..

[49] Krithivas A., Young D.B., Liao G., Greene D., Hayward S.D. (2000). Human herpesvirus 8 LANA interacts with proteins of the mSin3 corepressor complex and negatively regulates Epstein-Barr virus gene expression in dually infected PEL cells. J. Virol..

[50] Dittmer D.P., Damania B. (2016). Kaposi sarcoma-associated herpesvirus: Immunobiology, oncogenesis, and therapy. J. Clin. Investig..

[51] Shin Y.C., Nakamura H., Liang X., Feng P., Chang H., Kowalik T.F., Jung J.U. (2006). Inhibition of the ATM/p53 signal transduction pathway by Kaposi’s sarcoma-associated herpesvirus interferon regulatory factor 1. J. Virol..

[52] Seo T., Park J., Choe J. (2005). Kaposi’s sarcoma-associated herpesvirus viral IFN regulatory factor 1 inhibits transforming growth factor-beta signaling. Cancer Res..

[53] Ballestas M.E., Chatis P.A., Kaye K.M. (1999). Efficient persistence of extrachromosomal KSHV DNA mediated by latency-associated nuclear antigen. Science.

[54] Ballestas M.E., Kaye K.M. (2001). Kaposi’s sarcoma-associated herpesvirus latency-associated nuclear antigen 1 mediates episome persistence through cis-acting terminal repeat (tr) sequence and specifically binds tr DNA. J. Virol..

[55] Zhang Y.J., Wang K.Y., Stein D.A., Patel D., Watkins R., Moulton H.M., Iversen P.L., Matson D.O. (2007). Inhibition of replication and transcription activator and latency-associated nuclear antigen of Kaposi’s sarcoma-associated herpesvirus by morpholino oligomers. Antivir. Res..

[56] Skalsky R.L., Hu J., Renne R. (2007). Analysis of viral cis elements conferring Kaposi’s sarcoma-associated herpesvirus episome partitioning and maintenance. J. Virol..

[57] Keller S.A., Hernandez-Hopkins D., Vider J., Ponomarev V., Hyjek E., Schattner E.J., Cesarman E. (2006). NF-kappaB is essential for the progression of KSHV- and EBV-infected lymphomas in vivo. Blood.

[58] Keller S.A., Schattner E.J., Cesarman E. (2000). Inhibition of NF-kappaB induces apoptosis of KSHV-infected primary effusion lymphoma cells. Blood.

[59] Maecker H.T., McCoy J.P., Consortium F.H.I., Amos M., Elliott J., Gaigalas A., Wang L., Aranda R., Banchereau J., Boshoff C. (2010). A model for harmonizing flow cytometry in clinical trials. Nat. Immunol..

[60] Chaudhary P.M., Jasmin A., Eby M.T., Hood L. (1999). Modulation of the NF-kappa B pathway by virally encoded death effector domains-containing proteins. Oncogene.

[61] Labo N., Marshall V., Miley W., Davis E., McCann B., Stolka K.B., Ndom P., Hemingway-Foday J.J., Abassora M., Newton R. (2019). Mutual detection of Kaposi’s sarcoma-associated herpesvirus and Epstein-Barr virus in blood and saliva of cameroonians with and without Kaposi’s sarcoma. Int. J. Cancer.

[62] Sallah N., Miley W., Labo N., Carstensen T., Fatumo S., Gurdasani D., Pollard M.O., Dilthey A.T., Mentzer A.J., Marshall V. (2020). Distinct genetic architectures and environmental factors associate with host response to the gamma2-herpesvirus infections. Nat. Commun..

[63] Manners O., Murphy J.C., Coleman A., Hughes D.J., Whitehouse A. (2018). Contribution of the KSHV and EBV lytic cycles to tumourigenesis. Curr. Opin. Virol..

[64] Ma S.D., Hegde S., Young K.H., Sullivan R., Rajesh D., Zhou Y., Jankowska-Gan E., Burlingham W.J., Sun X., Gulley M.L. (2011). A new model of Epstein-Barr virus infection reveals an important role for early lytic viral protein expression in the development of lymphomas. J. Virol..

[65] Gloghini A., Volpi C.C., Gualeni A.V., Dolcetti R., Bongarzone I., de Paoli P., Carbone A. (2017). Multiple viral infections in primary effusion lymphoma: A model of viral cooperation in lymphomagenesis. Expert Rev. Hematol..

[66] Riva G., Lagreca I., Mattiolo A., Belletti D., Lignitto L., Barozzi P., Ruozi B., Vallerini D., Quadrelli C., Corradini G. (2015). Antineoplastic effects of liposomal short interfering RNA treatment targeting BLIMP1/PRDM1 in primary effusion lymphoma. Haematologica.

[67] Choi U.Y., Park A., Jung J.U. (2017). Double the trouble when herpesviruses join hands. Cell Host Microbe.

[68] Antsiferova O., Müller A., Rämer P., Chijioke O., Chatterjee B., Raykova A., Planas R., Sospedra M., Shumilov A., Tsai M.H. (2014). Adoptive transfer of EBV specific CD8^+^ T cell clones can transiently control EBV infection in humanized mice. PLoS Pathog..

[69] Bristol J.A., Djavadian R., Albright E.R., Coleman C.B., Ohashi M., Hayes M., Romero-Masters J.C., Barlow E.A., Farrell P.J., Rochford R. (2018). A cancer-associated Epstein-Barr virus BZLF1 promoter variant enhances lytic infection. PLoS Pathog..

[70] Münz C. (2021). The role of lytic infection for lymphomagenesis of human gamma-herpesviruses. Front. Cell Infect. Microbiol..

[71] McKenzie J., El-Guindy A. (2015). Epstein-Barr virus lytic cycle reactivation. Curr. Top. Microbiol. Immunol..

[72] Hong G.K., Gulley M.L., Feng W.H., Delecluse H.J., Holley-Guthrie E., Kenney S.C. (2005). Epstein-Barr virus lytic infection contributes to lymphoproliferative disease in a SCID mouse model. J. Virol..

[73] Jones R.J., Seaman W.T., Feng W.H., Barlow E., Dickerson S., Delecluse H.J., Kenney S.C. (2007). Roles of lytic viral infection and IL-6 in early versus late passage lymphoblastoid cell lines and EBV-associated lymphoproliferative disease. Int. J. Cancer.

[74] Mahot S., Sergeant A., Drouet E., Gruffat H. (2003). A novel function for the Epstein-Barr virus transcription factor EB1/Zta: Induction of transcription of the hIL-10 gene. J. Gen. Virol..

[75] Habib M., Buisson M., Lupo J., Agbalika F., Socie G., Germi R., Baccard M., Imbert-Marcille B.M., Dantal J., Morand P. (2017). Lytic EBV infection investigated by detection of soluble Epstein-Barr virus Zebra in the serum of patients with PTLD. Sci. Rep..

[76] Germini D., Sall F.B., Shmakova A., Wiels J., Dokudovskaya S., Drouet E., Vassetzky Y. (2020). Oncogenic properties of the EBV Zebra protein. Cancers.

[77] Okuno Y., Murata T., Sato Y., Muramatsu H., Ito Y., Watanabe T., Okuno T., Murakami N., Yoshida K., Sawada A. (2019). Defective Epstein-Barr virus in chronic active infection and haematological malignancy. Nat. Microbiol..

[78] Arvey A., Ojesina A.I., Pedamallu C.S., Ballon G., Jung J., Duke F., Leoncini L., de Falco G., Bressman E., Tam W. (2015). The tumor virus landscape of AIDS-related lymphomas. Blood.

[79] Casagrande N., Borghese C., Visser L., Mongiat M., Colombatti A., Aldinucci D. (2019). CCR5 antagonism by maraviroc inhibits Hodgkin lymphoma microenvironment interactions and xenograft growth. Haematologica.

[80] Walens A., DiMarco A.V., Lupo R., Kroger B.R., Damrauer J.S., Alvarez J.V. (2019). CCL5 promotes breast cancer recurrence through macrophage recruitment in residual tumors. eLife.

[81] Jochum S., Moosmann A., Lang S., Hammerschmidt W., Zeidler R. (2012). The EBV immunoevasins vIL-10 and BNLF2a protect newly infected B cells from immune recognition and elimination. PLoS Pathog..

[82] Jones K.D., Aoki Y., Chang Y., Moore P.S., Yarchoan R., Tosato G. (1999). Involvement of interleukin-10 (IL-10) and viral IL-6 in the spontaneous growth of Kaposi’s sarcoma herpesvirus-associated infected primary effusion lymphoma cells. Blood.

[83] Rosean T.R., Holman C.J., Tompkins V.S., Jing X., Krasowski M.D., Rose-John S., Janz S. (2016). KSHV-encoded vIL-6 collaborates with deregulated c-Myc to drive plasmablastic neoplasms in mice. Blood Cancer J..

[84] Suthaus J., Stuhlmann-Laeisz C., Tompkins V.S., Rosean T.R., Klapper W., Tosato G., Janz S., Scheller J., Rose-John S. (2012). HHV-8-encoded viral IL-6 collaborates with mouse IL-6 in the development of multicentric Castleman disease in mice. Blood.

[85] Sakakibara S., Tosato G. (2011). Viral interleukin-6: Role in Kaposi’s sarcoma-associated herpesvirus: Associated malignancies. J. Interferon Cytokine Res..

[86] Sakakibara S., Tosato G. (2014). Contribution of viral mimics of cellular genes to KSHV infection and disease. Viruses.

[87] Schulz T.F., Cesarman E. (2015). Kaposi sarcoma-associated herpesvirus: Mechanisms of oncogenesis. Curr. Opin. Virol..

[88] Pantanowitz L., Carbone A., Dolcetti R. (2015). Microenvironment and HIV-related lymphomagenesis. Semin. Cancer Biol..

[89] Chatterjee M., Osborne J., Bestetti G., Chang Y., Moore P.S. (2002). Viral IL-6-induced cell proliferation and immune evasion of interferon activity. Science.

[90] Punj V., Matta H., Schamus S., Yang T., Chang Y., Chaudhary P.M. (2009). Induction of CCL20 production by Kaposi sarcoma-associated herpesvirus: Role of viral FLICE inhibitory protein K13-induced NF-kappaB activation. Blood.

[91] Anders P.M., Montgomery N.D., Montgomery S.A., Bhatt A.P., Dittmer D.P., Damania B. (2018). Human herpesvirus-encoded kinase induces B cell lymphomas in vivo. J. Clin. Investig..

[92] Prakash O., Tang Z.Y., Peng X., Coleman R., Gill J., Farr G., Samaniego F. (2002). Tumorigenesis and aberrant signaling in transgenic mice expressing the human herpesvirus-8 K1 gene. J. Natl. Cancer Inst..

[93] Prakash O., Swamy O.R., Peng X., Tang Z.Y., Li L., Larson J.E., Cohen J.C., Gill J., Farr G., Wang S. (2005). Activation of SRC kinase Lyn by the Kaposi sarcoma-associated herpesvirus K1 protein: Implications for lymphomagenesis. Blood.

[94] Wang L., Wakisaka N., Tomlinson C.C., DeWire S.M., Krall S., Pagano J.S., Damania B. (2004). The Kaposi’s sarcoma-associated herpesvirus (KSHV/HHV-8) k1 protein induces expression of angiogenic and invasion factors. Cancer Res..

[95] Lee B.S., Lee S.H., Feng P., Chang H., Cho N.H., Jung J.U. (2005). Characterization of the Kaposi’s sarcoma-associated herpesvirus K1 signalosome. J. Virol..

[96] Cannon M.L., Cesarman E. (2004). The KSHV G protein-coupled receptor signals via multiple pathways to induce transcription factor activation in primary effusion lymphoma cells. Oncogene.

[97] Montaner S., Sodhi A., Molinolo A., Bugge T.H., Sawai E.T., He Y., Li Y., Ray P.E., Gutkind J.S. (2003). Endothelial infection with KSHV genes in vivo reveals that vGPCR initiates Kaposi’s sarcomagenesis and can promote the tumorigenic potential of viral latent genes. Cancer Cell.

[98] Nador R.G., Milligan L.L., Flore O., Wang X., Arvanitakis L., Knowles D.M., Cesarman E. (2001). Expression of Kaposi’s sarcoma-associated herpesvirus G protein-coupled receptor monocistronic and bicistronic transcripts in primary effusion lymphomas. Virology.

[99] Guo H.G., Sadowska M., Reid W., Tschachler E., Hayward G., Reitz M. (2003). Kaposi’s sarcoma-like tumors in a human herpesvirus 8 ORF74 transgenic mouse. J. Virol..

[100] Schwarz M., Murphy P.M. (2001). Kaposi’s sarcoma-associated herpesvirus G protein-coupled receptor constitutively activates NF-kappa B and induces proinflammatory cytokine and chemokine production via a c-terminal signaling determinant. J. Immunol..

[101] Jiang Y., Xu D., Zhao Y., Zhang L. (2008). Mutual inhibition between Kaposi’s sarcoma-associated herpesvirus and Epstein-Barr virus lytic replication initiators in dually-infected primary effusion lymphoma. PLoS ONE.

[102] Bentz G.L., Bheda-Malge A., Wang L., Shackelford J., Damania B., Pagano J.S. (2014). KSHV LANA and EBV LMP1 induce the expression of UCH-L1 following viral transformation. Virology.

[103] Sandhu P.K., Damania B. (2022). The regulation of KSHV lytic reactivation by viral and cellular factors. Curr. Opin. Virol..

[104] Lünemann A., Rowe M., Nadal D. (2015). Innate immune recognition of EBV. Curr. Top. Microbiol. Immunol..

[105] Lange P.T., White M.C., Damania B. (2022). Activation and evasion of innate immunity by gammaherpesviruses. J. Mol. Biol..

[106] Broussard G., Damania B. (2019). KSHV: Immune modulation and immunotherapy. Front. Immunol..

[107] Gujer C., Murer A., Muller A., Vanoaica D., Sutter K., Jacque E., Fournier N., Kalchschmidt J., Zbinden A., Capaul R. (2019). Plasmacytoid dendritic cells respond to Epstein-Barr virus infection with a distinct type I interferon subtype profile. Blood Adv..

[108] Casanova J.L., Abel L. (2013). The genetic theory of infectious diseases: A brief history and selected illustrations. Annu. Rev. Genom. Hum. Genet..

[109] Casanova J.L., Abel L., Quintana-Murci L. (2011). Human TLRs and IL-1Rs in host defense: Natural insights from evolutionary, epidemiological, and clinical genetics. Annu. Rev. Immunol..

[110] Sancho-Shimizu V., Perez de Diego R., Jouanguy E., Zhang S.Y., Casanova J.L. (2011). Inborn errors of anti-viral interferon immunity in humans. Curr. Opin. Virol..

[111] Giardino G., Cirillo E., Gallo V., Esposito T., Fusco F., Conte M.I., Quinti I., Ursini M.V., Carsetti R., Pignata C. (2015). B cells from nuclear factor kB essential modulator deficient patients fail to differentiate to antibody secreting cells in response to TLR9 ligand. Clin. Immunol..

[112] Panikkar A., Smith C., Hislop A., Tellam N., Dasari V., Hogquist K.A., Wykes M., Moss D.J., Rickinson A., Balfour H.H. (2015). Cytokine-mediated loss of blood dendritic cells during Epstein-Barr virus-associated acute infectious mononucleosis: Implication for immune dysregulation. J. Infect. Dis..

[113] Dunmire S.K., Grimm J.M., Schmeling D.O., Balfour H.H., Hogquist K.A. (2015). The incubation period of primary Epstein-Barr virus infection: Viral dynamics and immunologic events. PLoS Pathog..

[114] Van Gent M., Griffin B.D., Berkhoff E.G., van Leeuwen D., Boer I.G., Buisson M., Hartgers F.C., Burmeister W.P., Wiertz E.J., Ressing M.E. (2011). EBV lytic-phase protein BGLF5 contributes to TLR9 downregulation during productive infection. J. Immunol..

[115] Lefort S., Soucy-Faulkner A., Grandvaux N., Flamand L. (2007). Binding of Kaposi’s sarcoma-associated herpesvirus K-bZIP to interferon-responsive factor 3 elements modulates antiviral gene expression. J. Virol..

[116] Cloutier N., Flamand L. (2010). Kaposi sarcoma-associated herpesvirus latency-associated nuclear antigen inhibits interferon (IFN) beta expression by competing with IFN regulatory factor-3 for binding to IFNß promoter. J. Biol. Chem..

[117] Haneklaus M., Gerlic M., Kurowska-Stolarska M., Rainey A.A., Pich D., McInnes I.B., Hammerschmidt W., O’Neill L.A., Masters S.L. (2012). Cutting edge: MiR-223 and EBV miR-BART15 regulate the NLRP3 inflammasome and IL-1beta production. J. Immunol..

[118] Hooykaas M.J.G., van Gent M., Soppe J.A., Kruse E., Boer I.G.J., van Leenen D., Groot Koerkamp M.J.A., Holstege F.C.P., Ressing M.E., Wiertz E. (2017). EBV microRNA BART16 suppresses type I IFN signaling. J. Immunol..

[119] Lu Y., Qin Z., Wang J., Zheng X., Lu J., Zhang X., Wei L., Peng Q., Zheng Y., Ou C. (2017). Epstein-Barr virus miR-BART6-3p inhibits the RIG-I pathway. J. Innate. Immun..

[120] Abend J.R., Ramalingam D., Kieffer-Kwon P., Uldrick T.S., Yarchoan R., Ziegelbauer J.M. (2012). Kaposi’s sarcoma-associated herpesvirus microRNAs target IRAK1 and MyD88, two components of the toll-like receptor/interleukin-1R signaling cascade, to reduce inflammatory-cytokine expression. J. Virol..

[121] Gregory S.M., Davis B.K., West J.A., Taxman D.J., Matsuzawa S., Reed J.C., Ting J.P., Damania B. (2011). Discovery of a viral NLR homolog that inhibits the inflammasome. Science.

[122] Golas G., Jang S.J., Naik N.G., Alonso J.D., Papp B., Toth Z. (2020). Comparative analysis of the viral interferon regulatory factors of KSHV for their requisite for virus production and inhibition of the type I interferon pathway. Virology.

[123] Baresova P., Pitha P.M., Lubyova B. (2013). Distinct roles of Kaposi’s sarcoma-associated herpesvirus-encoded viral interferon regulatory factors in inflammatory response and cancer. J. Virol..

[124] Valentine R., Dawson C.W., Hu C., Shah K.M., Owen T.J., Date K.L., Maia S.P., Shao J., Arrand J.R., Young L.S. (2010). Epstein-Barr virus-encoded EBNA1 inhibits the canonical NF-kappaB pathway in carcinoma cells by inhibiting IKK phosphorylation. Mol. Cancer.

[125] Chen T., Wang Y., Xu Z., Zou X., Wang P., Ou X., Li Y., Peng T., Chen D., Li M. (2019). Epstein-Barr virus tegument protein BGLF2 inhibits NF-kappaB activity by preventing p65 Ser536 phosphorylation. FASEB J..

[126] Wang J.T., Doong S.L., Teng S.C., Lee C.P., Tsai C.H., Chen M.R. (2009). Epstein-Barr virus BGLF4 kinase suppresses the interferon regulatory factor 3 signaling pathway. J. Virol..

[127] Saito S., Murata T., Kanda T., Isomura H., Narita Y., Sugimoto A., Kawashima D., Tsurumi T. (2013). Epstein-Barr virus deubiquitinase downregulates TRAF6-mediated NF-kappaB signaling during productive replication. J. Virol..

[128] Yu Y., Wang S.E., Hayward G.S. (2005). The KSHV immediate-early transcription factor Rta encodes ubiquitin E3 ligase activity that targets IRF7 for proteosome-mediated degradation. Immunity.

[129] Zhu F.X., King S.M., Smith E.J., Levy D.E., Yuan Y. (2002). A Kaposi’s sarcoma-associated herpesviral protein inhibits virus-mediated induction of type I interferon by blocking IRF-7 phosphorylation and nuclear accumulation. Proc. Natl. Acad. Sci. USA.

[130] Sathish N., Zhu F.X., Golub E.E., Liang Q., Yuan Y. (2011). Mechanisms of autoinhibition of IRF-7 and a probable model for inactivation of IRF-7 by Kaposi’s sarcoma-associated herpesvirus protein ORF45. J. Biol. Chem..

[131] Damania B., Münz C. (2019). Immunodeficiencies that predispose to pathologies by human oncogenic gamma-herpesviruses. FEMS Microbiol. Rev..

[132] Azzi T., Lünemann A., Murer A., Ueda S., Beziat V., Malmberg K.J., Staubli G., Gysin C., Berger C., Münz C. (2014). Role for early-differentiated natural killer cells in infectious mononucleosis. Blood.

[133] Balfour H.H., Odumade O.A., Schmeling D.O., Mullan B.D., Ed J.A., Knight J.A., Vezina H.E., Thomas W., Hogquist K.A. (2013). Behavioral, virologic, and immunologic factors associated with acquisition and severity of primary Epstein-Barr virus infection in university students. J. Infect. Dis..

[134] Williams H., McAulay K., Macsween K.F., Gallacher N.J., Higgins C.D., Harrison N., Swerdlow A.J., Crawford D.H. (2005). The immune response to primary EBV infection: A role for natural killer cells. Br. J. Haematol..

[135] Williams L.R., Quinn L.L., Rowe M., Zuo J. (2016). Induction of the lytic cycle sensitizes Epstein-Barr virus-infected B cells to NK cell killing that is counteracted by virus-mediated NK cell evasion mechanisms in the late lytic cycle. J. Virol..

[136] Pappworth I.Y., Wang E.C., Rowe M. (2007). The switch from latent to productive infection in Epstein-Barr virus-infected B cells is associated with sensitization to NK cell killing. J. Virol..

[137] Chijioke O., Muller A., Feederle R., Barros M.H., Krieg C., Emmel V., Marcenaro E., Leung C.S., Antsiferova O., Landtwing V. (2013). Human natural killer cells prevent infectious mononucleosis features by targeting lytic Epstein-Barr virus infection. Cell Rep..

[138] Alari-Pahissa E., Ataya M., Moraitis I., Campos-Ruiz M., Altadill M., Muntasell A., Moles A., Lopez-Botet M. (2021). NK cells eliminate Epstein-Barr virus bound to B cells through a specific antibody-mediated uptake. PLoS Pathog..

[139] Lam J.K.P., Azzi T., Hui K.F., Wong A.M.G., McHugh D., Caduff N., Chan K.H., Münz C., Chiang A.K.S. (2020). Co-infection of cytomegalovirus and Epstein-Barr virus diminishes the frequency of CD56^dim^NKG2A^+^KIR^−^ NK cells and contributes to suboptimal control of EBV in immunosuppressed children with post-transplant lymphoproliferative disorder. Front. Immunol..

[140] Beldi-Ferchiou A., Lambert M., Dogniaux S., Vely F., Vivier E., Olive D., Dupuy S., Levasseur F., Zucman D., Lebbe C. (2016). PD-1 mediates functional exhaustion of activated NK cells in patients with Kaposi sarcoma. Oncotarget.

[141] Dupuy S., Lambert M., Zucman D., Choukem S.P., Tognarelli S., Pages C., Lebbe C., Caillat-Zucman S. (2012). Human herpesvirus 8 (HHV8) sequentially shapes the NK cell repertoire during the course of asymptomatic infection and Kaposi sarcoma. PLoS Pathog..

[142] Münz C. (2022). Natural killer cell responses to human oncogenic gamma-herpesvirus infections. Semin. Immunol..

[143] Thomas M., Boname J.M., Field S., Nejentsev S., Salio M., Cerundolo V., Wills M., Lehner P.J. (2008). Down-regulation of NKG2D and NKp80 ligands by Kaposi’s sarcoma-associated herpesvirus K5 protects against NK cell cytotoxicity. Proc. Natl. Acad. Sci. USA.

[144] Gabaev I., Williamson J.C., Crozier T.W.M., Schulz T.F., Lehner P.J. (2020). Quantitative proteomics analysis of lytic KSHV infection in human endothelial cells reveals targets of viral immune modulation. Cell Rep..

[145] Brulois K., Toth Z., Wong L.Y., Feng P., Gao S.J., Ensser A., Jung J.U. (2014). Kaposi’s sarcoma-associated herpesvirus K3 and K5 ubiquitin E3 ligases have stage-specific immune evasion roles during lytic replication. J. Virol..

[146] Yamin R., Kaynan N.S., Glasner A., Vitenshtein A., Tsukerman P., Bauman Y., Ophir Y., Elias S., Bar-On Y., Gur C. (2013). The viral KSHV chemokine vMIP-II inhibits the migration of naive and activated human NK cells by antagonizing two distinct chemokine receptors. PLoS Pathog..

[147] Nachmani D., Stern-Ginossar N., Sarid R., Mandelboim O. (2009). Diverse herpesvirus microRNAs target the stress-induced immune ligand MICB to escape recognition by natural killer cells. Cell Host Microbe.

[148] Münz C. (2020). Cytotoxicity in Epstein Barr virus specific immune control. Curr. Opin. Virol..

[149] Crawford D.H., Macsween K.F., Higgins C.D., Thomas R., McAulay K., Williams H., Harrison N., Reid S., Conacher M., Douglas J. (2006). A cohort study among university students: Identification of risk factors for Epstein-Barr virus seroconversion and infectious mononucleosis. Clin. Infect. Dis..

[150] Callan M.F., Tan L., Annels N., Ogg G.S., Wilson J.D., O’Callaghan C.A., Steven N., McMichael A.J., Rickinson A.B. (1998). Direct visualization of antigen-specific CD8^+^ T cells during the primary immune response to Epstein-Barr virus in vivo. J. Exp. Med..

[151] Catalina M.D., Sullivan J.L., Bak K.R., Luzuriaga K. (2001). Differential evolution and stability of epitope-specific CD8^+^ T cell responses in EBV infection. J. Immunol..

[152] Pudney V.A., Leese A.M., Rickinson A.B., Hislop A.D. (2005). CD8^+^ immunodominance among Epstein-Barr virus lytic cycle antigens directly reflects the efficiency of antigen presentation in lytically infected cells. J. Exp. Med..

[153] Woodberry T., Suscovich T.J., Henry L.M., Davis J.K., Frahm N., Walker B.D., Scadden D.T., Wang F., Brander C. (2005). Differential targeting and shifts in the immunodominance of Epstein-Barr virus—specific CD8 and CD4 T cell responses during acute and persistent infection. J. Infect. Dis..

[154] Hislop A.D., Annels N.E., Gudgeon N.H., Leese A.M., Rickinson A.B. (2002). Epitope-specific evolution of human CD8^+^ T cell responses from primary to persistent phases of Epstein-Barr virus infection. J. Exp. Med..

[155] Forrest C., Hislop A.D., Rickinson A.B., Zuo J. (2018). Proteome-wide analysis of CD8^+^ T cell responses to EBV reveals differences between primary and persistent infection. PLoS Pathog..

[156] Abbott R.J., Quinn L.L., Leese A.M., Scholes H.M., Pachnio A., Rickinson A.B. (2013). CD8^+^ T cell responses to lytic EBV infection: Late antigen specificities as subdominant components of the total response. J. Immunol..

[157] Orlova N., Wang F., Fogg M.H. (2011). Persistent infection drives the development of CD8^+^ T cells specific for late lytic infection antigens in lymphocryptovirus-infected macaques and Epstein-Barr virus-infected humans. J. Virol..

[158] Stowe R.P., Kozlova E.V., Yetman D.L., Walling D.M., Goodwin J.S., Glaser R. (2007). Chronic herpesvirus reactivation occurs in aging. Exp. Gerontol..

[159] Catalina M.D., Sullivan J.L., Brody R.M., Luzuriaga K. (2002). Phenotypic and functional heterogeneity of EBV epitope-specific CD8^+^ T cells. J. Immunol..

[160] Long H.M., Chagoury O.L., Leese A.M., Ryan G.B., James E., Morton L.T., Abbott R.J., Sabbah S., Kwok W., Rickinson A.B. (2013). MHC II tetramers visualize human CD4^+^ T cell responses to Epstein-Barr virus infection and demonstrate atypical kinetics of the nuclear antigen EBNA1 response. J. Exp. Med..

[161] Tamura Y., Yamane K., Kawano Y., Bullinger L., Wirtz T., Weber T., Sander S., Ohki S., Kitajima Y., Okada S. (2022). Concomitant cytotoxic effector differentiation of CD4^+^ and CD8^+^ T cells in response to EBV-infected B cells. Cancers.

[162] Meckiff B.J., Ladell K., McLaren J.E., Ryan G.B., Leese A.M., James E.A., Price D.A., Long H.M. (2019). Primary EBV infection induces an acute wave of activated antigen-specific cytotoxic CD4^+^ T cells. J. Immunol..

[163] Münz C., Bickham K.L., Subklewe M., Tsang M.L., Chahroudi A., Kurilla M.G., Zhang D., O’Donnell M., Steinman R.M. (2000). Human CD4^+^ T lymphocytes consistently respond to the latent Epstein-Barr virus nuclear antigen EBNA1. J. Exp. Med..

[164] Long H.M., Meckiff B.J., Taylor G.S. (2019). The T-cell response to Epstein-Barr virus-new tricks from an old dog. Front. Immunol..

[165] Jayasooriya S., de Silva T.I., Njie-jobe J., Sanyang C., Leese A.M., Bell A.I., McAulay K.A., Yanchun P., Long H.M., Dong T. (2015). Early virological and immunological events in asymptomatic Epstein-Barr virus infection in African children. PLoS Pathog..

[166] Grant M.L., Bollard C.M. (2018). Cell therapies for hematological malignancies: Don’t forget non-gene-modified T cells!. Blood Rev..

[167] McHugh D., Caduff N., Murer A., Engelmann C., Deng Y., Zdimerova H., Zens K., Chijioke O., Münz C. (2019). Infection and immune control of human oncogenic gamma-herpesviruses in humanized mice. Philos. Trans. R. Soc. Lond. B Biol. Sci..

[168] Nalwoga A., Roshan R., Moore K., Marshall V., Miley W., Labo N., Nakibuule M., Cose S., Rochford R., Newton R. (2021). Kaposi’s sarcoma-associated herpesvirus T cell responses in HIV seronegative individuals from rural Uganda. Nat. Commun..

[169] Roshan R., Labo N., Trivett M., Miley W., Marshall V., Coren L., Cornejo Castro E.M., Perez H., Holdridge B., Davis E. (2017). T-cell responses to KSHV infection: A systematic approach. Oncotarget.

[170] Robey R.C., Mletzko S., Gotch F.M. (2010). The T-cell immune response against Kaposi’s sarcoma-associated herpesvirus. Adv. Virol..

[171] Robey R.C., Lagos D., Gratrix F., Henderson S., Matthews N.C., Vart R.J., Bower M., Boshoff C., Gotch F.M. (2009). The CD8 and CD4 T-cell response against Kaposi’s sarcoma-associated herpesvirus is skewed towards early and late lytic antigens. PLoS ONE.

[172] Hislop A.D., Ressing M.E., van Leeuwen D., Pudney V.A., Horst D., Koppers-Lalic D., Croft N.P., Neefjes J.J., Rickinson A.B., Wiertz E.J. (2007). A CD8^+^ T cell immune evasion protein specific to Epstein-Barr virus and its close relatives in old world primates. J. Exp. Med..

[173] Wang Q.J., Jenkins F.J., Jacobson L.P., Kingsley L.A., Day R.D., Zhang Z.W., Meng Y.X., Pellett P.E., Kousoulas K.G., Baghian A. (2001). Primary human herpesvirus 8 infection generates a broadly specific CD8^+^ T-cell response to viral lytic cycle proteins. Blood.

[174] Andreoni M., Sarmati L., Nicastri E., El Sawaf G., El Zalabani M., Uccella I., Bugarini R., Parisi S.G., Rezza G. (2002). Primary human herpesvirus 8 infection in immunocompetent children. JAMA.

[175] Chen R.L., Lin J.C., Wang P.J., Lee C.P., Hsu Y.H. (2004). Human herpesvirus 8-related childhood mononucleosis: A series of three cases. Pediatr. Infect. Dis. J..

[176] Trovato R., Luppi M., Barozzi P., Da Prato L., Maiorana A., Lico S., Marasca R., Torricelli P., Torelli G., Ceccherini-Nelli L. (1999). Cellular localization of human herpesvirus 8 in nonneoplastic lymphadenopathies and chronic interstitial pneumonitis by in situ polymerase chain reaction studies. J. Hum. Virol..

[177] Barcy S., de Rosa S.C., Vieira J., Diem K., Ikoma M., Casper C., Corey L. (2008). Gamma delta^+^ T cells involvement in viral immune control of chronic human herpesvirus 8 infection. J. Immunol..

[178] Shrestha P., Davis D.A., Jaeger H.K., Stream A., Aisabor A.I., Yarchoan R. (2021). Pomalidomide restores immune recognition of primary effusion lymphoma through upregulation of ICAM-1 and B7-2. PLoS Pathog..

[179] Brander C., Suscovich T., Lee Y., Nguyen P.T., O’Connor P., Seebach J., Jones N.G., van Gorder M., Walker B.D., Scadden D.T. (2000). Impaired CTL recognition of cells latently infected with Kaposi’s sarcoma-associated herpes virus. J. Immunol..

[180] Usherwood E.J., Meadows S.K., Crist S.G., Bellfy S.C., Sentman C.L. (2005). Control of murine gammaherpesvirus infection is independent of NK cells. Eur. J. Immunol..

[181] Guihot A., Dupin N., Marcelin A.G., Gorin I., Bedin A.S., Bossi P., Galicier L., Oksenhendler E., Autran B., Carcelain G. (2006). Low T cell responses to human herpesvirus 8 in patients with AIDS-related and classic Kaposi sarcoma. J. Infect. Dis..

[182] Lambert M., Gannage M., Karras A., Abel M., Legendre C., Kerob D., Agbalika F., Girard P.M., Lebbe C., Caillat-Zucman S. (2006). Differences in the frequency and function of HHV8-specific CD8 T cells between asymptomatic HHV8 infection and Kaposi sarcoma. Blood.

[183] Lepone L., Rappocciolo G., Knowlton E., Jais M., Piazza P., Jenkins F.J., Rinaldo C.R. (2010). Monofunctional and polyfunctional CD8^+^ T cell responses to human herpesvirus 8 lytic and latency proteins. Clin. Vaccine Immunol..

[184] Guihot A., Oksenhendler E., Galicier L., Marcelin A.G., Papagno L., Bedin A.S., Agbalika F., Dupin N., Cadranel J., Autran B. (2008). Multicentric Castleman disease is associated with polyfunctional effector memory HHV-8-specific CD8^+^ T cells. Blood.

[185] Bihl F., Berger C., Chisholm J.V., Henry L.M., Bertisch B., Trojan A., Nadal D., Speck R.F., Flepp M., Brander C. (2009). Cellular immune responses and disease control in acute AIDS-associated Kaposi’s sarcoma. AIDS.

[186] Camcioglu Y., Picard C., Lacoste V., Dupuis S., Akcakaya N., Cokura H., Kaner G., Demirkesen C., Plancoulaine S., Emile J.F. (2004). HHV-8-associated Kaposi sarcoma in a child with IFNgammar1 deficiency. J. Pediatr..

[187] Aavikko M., Kaasinen E., Nieminen J.K., Byun M., Donner I., Mancuso R., Ferrante P., Clerici M., Brambilla L., Tourlaki A. (2015). Whole-genome sequencing identifies STAT4 as a putative susceptibility gene in classic Kaposi sarcoma. J. Infect. Dis..

[188] Ensoli B., Sgadari C., Barillari G., Sirianni M.C., Sturzl M., Monini P. (2001). Biology of Kaposi’s sarcoma. Eur. J. Cancer.

[189] Luttichau H.R., Lewis I.C., Gerstoft J., Schwartz T.W. (2001). The herpesvirus 8-encoded chemokine vMIP-II, but not the poxvirus-encoded chemokine MC148, inhibits the CCR10 receptor. Eur. J. Immunol..

[190] Pontejo S.M., Murphy P.M. (2017). Chemokines encoded by herpesviruses. J. Leukoc. Biol..

[191] Ngalamika O., Mukasine M.C., Kawimbe M., Vally F. (2021). Viral and immunological markers of HIV-associated Kaposi sarcoma recurrence. PLoS ONE.

[192] Matiza T., Boyd K.F., Lyall R.A., Kwon D.S., McGregor A.M., Fiorillo S., Campbell T.B., Borok M., Corleis B. (2021). Compartmentalized T cell profile in the lungs of patients with HIV-1-associated pulmonary Kaposi sarcoma. Medicine.

[193] Cohen J.I., Fauci A.S., Varmus H., Nabel G.J. (2011). Epstein-Barr virus: An important vaccine target for cancer prevention. Sci. Transl. Med..

[194] Wong Y., Meehan M.T., Burrows S.R., Doolan D.L., Miles J.J. (2022). Estimating the global burden of Epstein-Barr virus-related cancers. J. Cancer Res. Clin. Oncol..

[195] Barasa A.K., Ye P., Phelps M., Arivudainambi G.T., Tison T., Ogembo J.G. (2017). BALB/c mice immunized with a combination of virus-like particles incorporating Kaposi sarcoma-associated herpesvirus (KSHV) envelope glycoproteins gpK8.1, gB, and gH/gL induced comparable serum neutralizing antibody activity to UV-inactivated KSHV. Oncotarget.

[196] Mulama D.H., Mutsvunguma L.Z., Totonchy J., Ye P., Foley J., Escalante G.M., Rodriguez E., Nabiee R., Muniraju M., Wussow F. (2019). A multivalent Kaposi sarcoma-associated herpesvirus-like particle vaccine capable of eliciting high titers of neutralizing antibodies in immunized rabbits. Vaccine.

[197] Wei C.J., Bu W., Nguyen L.A., Batchelor J.D., Kim J., Pittaluga S., Fuller J.R., Nguyen H., Chou T.H., Cohen J.I. (2022). A bivalent Epstein-Barr virus vaccine induces neutralizing antibodies that block infection and confer immunity in humanized mice. Sci. Transl. Med..

[198] Moutschen M., Leonard P., Sokal E.M., Smets F., Haumont M., Mazzu P., Bollen A., Denamur F., Peeters P., Dubin G. (2007). Phase I/II studies to evaluate safety and immunogenicity of a recombinant gp350 Epstein-Barr virus vaccine in healthy adults. Vaccine.

[199] Ruiss R., Jochum S., Wanner G., Reisbach G., Hammerschmidt W., Zeidler R. (2011). A virus-like particle-based Epstein-Barr virus vaccine. J. Virol..

[200] Sokal E.M., Hoppenbrouwers K., Vandermeulen C., Moutschen M., Leonard P., Moreels A., Haumont M., Bollen A., Smets F., Denis M. (2007). Recombinant gp350 vaccine for infectious mononucleosis: A phase 2, randomized, double-blind, placebo-controlled trial to evaluate the safety, immunogenicity, and efficacy of an Epstein-Barr virus vaccine in healthy young adults. J. Infect. Dis..

[201] Latour S., Fischer A. (2019). Signaling pathways involved in the T-cell-mediated immunity against Epstein-Barr virus: Lessons from genetic diseases. Immunol. Rev..

[202] Taylor G.S., Haigh T.A., Gudgeon N.H., Phelps R.J., Lee S.P., Steven N.M., Rickinson A.B. (2004). Dual stimulation of Epstein-Barr virus (EBV)-specific CD4^+^- and CD8^+^-T-cell responses by a chimeric antigen construct: Potential therapeutic vaccine for EBV-positive nasopharyngeal carcinoma. J. Virol..

[203] Taylor G.S., Jia H., Harrington K., Lee L.W., Turner J., Ladell K., Price D.A., Tanday M., Matthews J., Roberts C. (2014). A recombinant modified vaccinia Ankara vaccine encoding Epstein-Barr virus (EBV) target antigens: A phase I trial in UK patients with EBV-positive cancer. Clin. Cancer Res..

[204] Hui E.P., Taylor G.S., Jia H., Ma B.B., Chan S.L., Ho R., Wong W.L., Wilson S., Johnson B.F., Edwards C. (2013). Phase I trial of recombinant modified vaccinia Ankara encoding Epstein-Barr viral tumor antigens in nasopharyngeal carcinoma patients. Cancer Res..

[205] Rühl J., Citterio C., Engelmann C., Haigh T.A., Dzionek A., Dreyer J.H., Khanna R., Taylor G.S., Wilson J.B., Leung C.S. (2019). Heterologous prime-boost vaccination protects from EBV antigen expressing lymphomas. J. Clin. Investig..

[206] Dunmire S.K., Verghese P.S., Balfour H.H. (2018). Primary Epstein-Barr virus infection. J. Clin. Virol..

[207] Rostgaard K., Balfour H.H., Jarrett R., Erikstrup C., Pedersen O., Ullum H., Nielsen L.P., Voldstedlund M., Hjalgrim H. (2019). Primary Epstein-Barr virus infection with and without infectious mononucleosis. PLoS ONE.

[208] Hjalgrim H., Askling J., Rostgaard K., Hamilton-Dutoit S., Frisch M., Zhang J.S., Madsen M., Rosdahl N., Konradsen H.B., Storm H.H. (2003). Characteristics of Hodgkin’s lymphoma after infectious mononucleosis. N. Engl. J. Med..

[209] Sundqvist E., Sundstrom P., Linden M., Hedstrom A.K., Aloisi F., Hillert J., Kockum I., Alfredsson L., Olsson T. (2012). Epstein-Barr virus and multiple sclerosis: Interaction with HLA. Genes Immun..

[210] Thacker E.L., Mirzaei F., Ascherio A. (2006). Infectious mononucleosis and risk for multiple sclerosis: A meta-analysis. Ann. Neurol..

[211] Attfield K.E., Jensen L.T., Kaufmann M., Friese M.A., Fugger L. (2022). The immunology of multiple sclerosis. Nat. Rev. Immunol..

[212] Bjornevik K., Cortese M., Healy B.C., Kuhle J., Mina M.J., Leng Y., Elledge S.J., Niebuhr D.W., Scher A.I., Munger K.L. (2022). Longitudinal analysis reveals high prevalence of Epstein-Barr virus associated with multiple sclerosis. Science.

